# Estimating the Attack Rate of Pregnancy-Associated Listeriosis during a Large Outbreak

**DOI:** 10.1155/2015/201479

**Published:** 2015-02-15

**Authors:** Maho Imanishi, Janell A. Routh, Marigny Klaber, Weidong Gu, Michelle S. Vanselow, Kelly A. Jackson, Loretta Sullivan-Chang, Gretchen Heinrichs, Neena Jain, Bernadette Albanese, William M. Callaghan, Barbara E. Mahon, Benjamin J. Silk

**Affiliations:** ^1^Division of Foodborne, Waterborne, and Environmental Diseases, Centers for Disease Control and Prevention, Atlanta, GA 30333, USA; ^2^Epidemic Intelligence Service, Scientific Education and Professional Development Program Office, Centers for Disease Control and Prevention, Atlanta, GA 30329, USA; ^3^El Paso County Public Health, Colorado Springs, CO 80907, USA; ^4^Denver Health and Hospital Authority, Denver, CO 80204, USA; ^5^Tri-County Health Department, Greenwood Village, CO 80111, USA; ^6^Division of Reproductive Health, Centers for Disease Control and Prevention, Atlanta, GA 30329, USA

## Abstract

*Background*. In 2011, a multistate outbreak of listeriosis linked to contaminated cantaloupes raised concerns that many pregnant women might have been exposed to *Listeria monocytogenes*. Listeriosis during pregnancy can cause fetal death, premature delivery, and neonatal sepsis and meningitis. Little information is available to guide healthcare providers who care for asymptomatic pregnant women with suspected *L. monocytogenes* exposure. *Methods*. We tracked pregnancy-associated listeriosis cases using reportable diseases surveillance and enhanced surveillance for fetal death using vital records and inpatient fetal deaths data in Colorado. We surveyed 1,060 pregnant women about symptoms and exposures. We developed three methods to estimate how many pregnant women in Colorado ate the implicated cantaloupes, and we calculated attack rates. *Results*. One laboratory-confirmed case of listeriosis was associated with pregnancy. The fetal death rate did not increase significantly compared to preoutbreak periods. Approximately 6,500–12,000 pregnant women in Colorado might have eaten the contaminated cantaloupes, an attack rate of ~1 per 10,000 exposed pregnant women. *Conclusions*. Despite many exposures, the risk of pregnancy-associated listeriosis was low. Our methods for estimating attack rates may help during future outbreaks and product recalls. Our findings offer relevant considerations for management of asymptomatic pregnant women with possible *L. monocytogenes* exposure.

## 1. Introduction

Invasive* L. monocytogenes* infection (listeriosis) is a rare but severe foodborne illness. Those at highest risk include older adults, immunocompromised persons, and pregnant women and their newborns. Listeriosis outbreaks and product recalls raise questions about medical management of these patients when exposure is suspected. However, the results of serological tests and stool cultures to screen for listeriosis have poor predictive value [1, 2]; furthermore, the relationship between exposure dose and illness is complex [3].

In the United States, about 17% of listeriosis cases that occur annually are associated with pregnancy and the incidence is ~3.4 reported cases per 100,000 pregnant women [4]. Maternal bacteremia typically presents as a febrile illness with nonspecific symptoms. Gastroenteritis or asymptomatic maternal infections also occur [5]. Complications including fetal death, preterm delivery, and neonatal sepsis, meningitis, and death may result when* L. monocytogenes* invades the placenta [6].

In 2011, a large outbreak of listeriosis linked to contaminated whole cantaloupe from a single farm in Colorado (Farm A) led to 147 confirmed cases in 28 states, including 33 deaths and 1 fetal loss [7]. Pregnant women accounted for <5% of reported cases (7 cases) [7]. In contrast, 12%–83% of cases were pregnancy-associated in other major North American outbreaks [8–14]. The relatively low proportion of pregnancy-associated cases during the 2011 outbreak was recognized early. However, the incubation period of listeriosis is longer for pregnancy-associated cases, and health officials feared a late surge [11, 15]. This prompted CDC and state and local health authorities to conduct a multifaceted investigation in Colorado to enhance surveillance and estimate the attack rate among pregnant women who ate the implicated cantaloupes.

## 2. Methods

### 2.1. Definitions

Pregnancy-associated cases were defined by isolation of* L. monocytogenes* from a clinical specimen collected from a pregnant woman, fetus, or an infant ≤31 days of age. The outbreak period was defined as August 2011–October 2011 [7].

### 2.2. Enhanced Surveillance

Listeriosis is reportable in Colorado; therefore, we searched the Colorado Department of Public Health and Environment (CDPHE) surveillance system for laboratory-confirmed cases. We enhanced surveillance by analyzing two data sources for increases in all-cause fetal deaths. First, we reviewed inpatient fetal deaths and births during January–October 2011 from nine hospitals in Denver and Colorado Springs, the cities reporting the most outbreak-related cases. We compared preoutbreak rates of inpatient fetal death (January–July 2011) to rates during the outbreak period. Second, we reviewed fetal deaths at ≥20 weeks of gestation and total births reported to CDPHE's vital records registry to compare the rates of fetal death in the outbreak period to historical (2006–2010) rates. To assess whether fetal death rates increased during the outbreak, we used Poisson regression to estimate relative rates (RRs) and 95% confidence intervals (95% CIs). We also reviewed medical records for inpatient fetal deaths at ≥20 weeks of gestation during the outbreak at three hospitals in a Colorado county with many outbreak-related cases. We abstracted data on maternal clinical presentation, placental pathology, fetal autopsy and pathology, and laboratory test results.

### 2.3. Survey of Pregnant Women

Pregnant women visiting 28 healthcare facilities (private clinics, community health clinics, and a county hospital) in eight Colorado cities were invited to participate in an anonymous survey that asked about general knowledge of listeriosis and the outbreak, history of consuming cantaloupe and other foods between August 1, 2011 and the survey date (September 27–October 14, 2011), and symptoms experienced during this period. We summarized response frequencies and calculated odds ratios (ORs) and 95% CIs for symptoms, comparing cantaloupe consumers with nonconsumers.

### 2.4. Estimation of Attack Rate

We estimated attack rates by dividing pregnancy-associated cases reported in Colorado by the estimated number of pregnant women who ate Farm A cantaloupe during the outbreak.

We assumed that all Farm A cantaloupes distributed in 2011 were contaminated to some extent. The first outbreak-related illness occurred shortly after harvest began, and extensive contamination at the processing facility was found during environmental and root-cause investigations of the farm [16]. Supporting our assumption, 17 (94%) of 18 Farm A cantaloupes collected from Colorado stores yielded an outbreak strain [7].

We estimated how many pregnant women (*N*_Preg_) were in Colorado for the 6-week exposure period, when Farm A cantaloupe was on the market in Colorado, by

(1) $$\begin{array}{c} N_{\text{Preg}}=\frac{N_{\text{Birth}}}{52}\left[40+5\right], \end{array}$$

where *N*_Birth_ are live births in 2011 in Colorado (65,511) (CDPHE vital records, unpublished data). *N*_Birth_ is divided by 52 to calculate average live births per week (i.e., approximating new pregnancies) and multiplied by 40 to estimate the average number of pregnant women in a week, because full-term pregnancy lasts about 40 weeks. By adding 5 weeks to the multiplier, we accounted for new pregnancies during the remaining 5 weeks of the exposure period. We estimated how many pregnant women ate the cantaloupe (*N*_Preg,Cant_) by

(2) $$\begin{array}{c} N_{\text{Preg},\text{Cant}}=N_{\text{Preg}}p_{\text{Preg},\text{Cant}}, \end{array}$$

where *p*_Preg,Cant_ is the proportion of pregnant women surveyed who reported eating cantaloupe.

We developed three methods—each based on different data and assumptions—to estimate how many pregnant women ate Farm A cantaloupe (*N*_Preg,A1–3_). In the first method

(3) $$\begin{array}{c} N_{\text{Preg},\text{A}1}=N_{\text{Preg},\text{Cant}}\frac{C_{\text{A},\text{Dist}}}{C_{\text{Cant},\text{CO}}} \end{array}$$

*C*_A,Dist_ is the weight of cantaloupe distributed nationally by Farm A in 2011 and *C*_Cant,CO_ is the weight of cantaloupe produced in Colorado in 2011. We assumed that all cantaloupe sold in Colorado during the exposure period was produced in Colorado and that Colorado cantaloupe farms distributed the same proportion of their harvest out of the state (i.e., the ratio of* distributed* to* produced* cantaloupe approximates the proportion of Farm A cantaloupe in Colorado).

The second method is

(4) $$\begin{array}{c} N_{\text{Preg},\text{A}2}=N_{\text{Preg},\text{Cant}}\frac{\sum_{i=1,\ldots ,n}\left(p_{\text{CantA},i}N_{\text{preg},i}\right)}{\sum_{i=1,\ldots ,n}N_{\text{preg},i}}, \end{array}$$

where *p*_Cant,A,*i*_ is the approximate proportion of Farm A cantaloupe available in *i* grocery store chain (*n* = 7) reported by pregnant women in the survey during the exposure period in Colorado. *N*_Preg,*i*_ is the number of pregnant women who reported purchasing cantaloupe at *i* grocery store chain. The number of pregnant women who ate cantaloupe was multiplied by grocery store chain-specific proportions of Farm A cantaloupe, weighted by the number of pregnant women who reported a purchase location. When the proportion of Farm A cantaloupe sold at a reported purchase location could not be obtained (e.g., farmer's market), the data were excluded. If a pregnant woman reported purchasing cantaloupe at multiple grocery store chains, the store with the highest proportion of Farm A cantaloupe was assumed to represent her purchase location.

The third method was

(5) $$\begin{array}{c} N_{\text{Preg},\text{A}3}=\frac{C_{\text{A},\text{CO}}p_{\text{Preg}}}{C_{\text{Cant},\text{Preg}}}, \end{array}$$

where *C*_A,CO_ is the number of Farm A cantaloupes distributed in Colorado (estimated by dividing the weight of distributed cantaloupe by an average weight of 15 Farm A cantaloupe melons collected for testing), *p*_Preg_ is the estimated proportion of pregnant Colorado residents, and *C*_Cant,Preg_ is the estimated number of cantaloupe eaten by each pregnant woman during the exposure period. Cantaloupe consumption was estimated by using the servings and amount per serving reported by pregnant women surveyed. We assumed that all distributed Farm A cantaloupes were eaten, that Colorado residents were equally likely to eat Farm A cantaloupe (no brand or store preference), and that Colorado residents were likely to eat the same amount of cantaloupe as pregnant women.

For all three methods, we estimated how many women were exposed and rounded to two significant figures; however, our estimations used exact numbers. This investigation was part of a nonresearch public health emergency response and therefore exempt from the CDC Institutional Review Board process.

## 3. Results

During the outbreak investigation conducted during August–October 2011, 40 outbreak-related cases of listeriosis were reported in Colorado. One (2.5%) case occurred in a pregnant woman; no neonatal cases were reported.

Among nine hospitals included in enhanced surveillance, the rate of inpatient fetal death during the outbreak was not significantly higher than the preoutbreak period (RR = 1.27, 95% CI: 0.93–1.71). Our review of vital records showed that the rate of fetal death per 1,000 live births was not significantly different during the outbreak compared to during the same months in 2006–2010 (RR = 0.85, 95% CI: 0.66–1.07). At three hospitals in a county with many outbreak-related cases, 28 fetal deaths due to any cause occurred. Placental histopathology results were available for 25 of these fetal deaths; none showed evidence of listeriosis. Other causes were identified for the two fetal deaths in which placental histopathology was not performed. In one fetal death in which the mother presented with nausea and vomiting but no fever, no further diagnostic evaluation was undertaken.

Among 1,060 pregnant women who completed some or all of the survey, 81% reported having heard about the listeriosis outbreak. However, few had contacted a healthcare provider (5%, 47/1,033) or the health department (1%, 8/1,035) with questions about listeriosis. When asked about their history of eating cantaloupe or other higher-risk foods between August 1 and the survey date, 37% reported eating or likely eating cantaloupe; most could not recall what brand they had eaten. Many pregnant women reported eating or likely eating other foods commonly associated with listeriosis, including turkey deli meat (54%) and soft cheese (39%). Although 22 (2%) of 883 pregnant women who responded to the question on symptoms reported fever, fever was not significantly associated with eating cantaloupe (OR = 1.3, 95% CI: 0.6–3.2). No respondents reported a diagnosis of listeriosis at the time of the survey.

Our estimates of attack rate ranged from 0.9 to 1.5 cases per 10,000 pregnant women who ate cantaloupes (Table 1). An estimated 57,000 pregnant women were in Colorado during the outbreak. By using three methods, we estimated that 6,500–12,000 pregnant women were exposed to Farm A cantaloupe. In (3), national distribution (*C*_A,Dist_) was reported as ~12.4 million pounds (FDA, unpublished data) and Colorado production (*C*_Cant,CO_) as ~40 million pounds [17]. Therefore, 6,500 pregnant women ate Farm A cantaloupe (*N*_preg,A1_). In (4), the proportion of Farm A cantaloupe sold at each grocery store chain (*p*_CantA,*i*_) ranged from 0-90% among the seven major chains. Of the 207 women who purchased cantaloupe in at least one chain, the number of pregnant women per chain (*N*_preg,*i*_) ranged from 6 to 85. By applying grocery store chain-specific weights, *N*_Preg,A2_ was estimated to be 12,000. In (5), the weight of Farm A cantaloupe distributed in Colorado was reportedly 4.6 million pounds. The average weight of 15 cantaloupes collected for testing was 4.14 pounds, making the total estimated Farm A cantaloupes distributed in Colorado (*C*_A,CO_ *p*_Preg_) 1.1 million (FDA, unpublished data). The proportion of pregnant women among Colorado residents (*p*_Preg_) was 1.1% [18], and the average amount eaten during the exposure period (*C*_Cant,Preg_) was 1.7 cantaloupes per pregnant woman, leading to an estimate (*N*_Preg,A3_) of 7,200.

## 4. Discussion

Despite widespread exposure to cantaloupes contaminated with* L. monocytogenes*, the attack rate of listeriosis among pregnant women in Colorado was low—about 1.0 case per 10,000 exposed pregnant women. The late surge in pregnancy-associated cases in Colorado feared by public health officials did not occur. To our knowledge, these are the first estimates of attack rates for pregnancy-associated listeriosis during a large-scale outbreak. When product distribution and retail data are available, our methods and findings may be useful during future* Listeria* outbreaks and product recalls. In this outbreak, they suggest that diagnostic testing to screen for infection and prophylactic treatment of asymptomatic pregnant women with possible* L. monocytogenes* exposure may have had limited utility.

Our finding that pregnant women were relatively unaffected during the outbreak was supported by several lines of evidence. CDPHE did not receive reports of invasive neonatal listeriosis. Neonatal listeriosis is unlikely to be missed because blood (and often cerebrospinal fluid) culture is routine in sick neonates. Also, our reviews of records showed no significant increase in fetal death rates attributable to listeriosis during the outbreak. Few pregnant women whom we surveyed reported fever, the most common symptom of listeriosis [5, 19]. More importantly, the proportion of pregnant women reporting fever was similar among those who did and did not report eating cantaloupe.

We compared our findings to the outbreak data for older adults, another group with a higher risk of listeriosis. A total of 32 listeriosis cases were reported among ~576,000 adults ≥65 years of age in Colorado during the outbreak [18]. Using these data, the attack rate estimate is 5.6 cases per 10,000 population. However, the actual attack rate would be substantially higher had we limited this estimate to older adults who consumed Farm A cantaloupe, a denominator directly comparable to that used for our estimate of 1.0 case per 10,000 for exposed pregnant women.

Many possibilities could explain the relatively few pregnancy-associated cases reported compared with older adults. Data from a 2006–2007 survey of the US population on foods eaten in the past 7 days indicate that cantaloupes were eaten more frequently by adults ≥65 years of age (56%) than by women of reproductive age (36%) in August and September (CDC, unpublished data).* Listeria* can multiply at refrigerator temperatures, so we speculate that if older adults handled cantaloupe differently and stored it longer, they might have been exposed to a higher dose than pregnant women [20]. However, bacterial enumeration data (e.g., colony forming units per gram of cantaloupe) were not available to characterize the role of dose. The outbreak was caused by multiple* L. monocytogenes* strains belonging to serotypes 1/2*a* and 1/2*b*, but serotype 4*b* is the most frequent serotype causing pregnancy-associated listeriosis [4]. The effects of* L. monocytogenes* virulence (or virulence attenuation) factors may differ between pregnant women and older adults, leading to differences in the risk of invasive disease following infection [21, 22]. We surveyed prenatal care providers in Colorado during the outbreak investigation and found that only 12 (6%) of 206 providers had prescribed prophylactic treatment. Thus, prophylactic treatment is an unlikely explanation for the low number of pregnancy-associated cases (CDC, unpublished data).

Attack rate estimates should be interpreted with caution because of this investigation's limitations. Pregnancy-associated listeriosis is rare, and a report of one additional case would have doubled our attack rate estimates. Pregnancy-associated cases resulting in early fetal death (at <20 weeks of gestation) may have been missed because cultures and histopathologic investigation often are not performed to determine etiology. We monitored the rate of all-cause fetal death as a proxy. However, neither the sensitivity nor the specificity of fetal death, as an indicator for detecting an increase in the rate of pregnancy-associated listeriosis, is optimal. Also, the strength of evidence for another cause of fetal death varied (e.g., identification of a chromosomal abnormality versus absence of infectious etiology in placental pathology). Further, we could only approximate how many pregnant women ate Farm A cantaloupe because most could not recall the brand. We did not know how many cantaloupes were sold in Colorado; when we asked women about cantaloupe they had eaten after August 1, we included some days after the cantaloupe was recalled on September 14, 2011. We used multiple simplifying assumptions to estimate women at risk, cantaloupe market distribution and purchases, and likelihood of cantaloupe consumption and contamination. Confidence intervals for attack rates could not be reliably calculated because the variance of several variables in these estimates could not be determined. Nevertheless, the fact that point estimates produced by the three methods were within an order of magnitude of each other is somewhat reassuring.

## 5. Conclusion

In September 2011, CDC rapidly convened several national experts in infectious diseases, obstetrics, and public health to develop a suggested framework for medical management of persons at elevated risk for invasive listeriosis who may have been exposed to* L. monocytogenes*. The experts concluded that neither diagnostic testing nor antimicrobial therapy (prophylactic treatment) is medically indicated for asymptomatic patients with possible exposure because the risk of developing an invasive disease is low. Subsequently, the American College of Obstetricians and Gynecologists (ACOG) published an obstetric practice committee opinion on management of pregnant women with presumptive exposure to* L. monocytogenes*, which concluded that “No testing, including blood and stool cultures, or treatment is indicated for an asymptomatic pregnant woman who reports consumption of a product that was recalled or implicated during an outbreak of listeria contamination.” [23]. Our evidence supports their opinions.

Medical management of pregnant women possibly exposed to* L. monocytogenes* should emphasize monitoring for symptoms consistent with listeriosis and set a low threshold for medical evaluation of those who are symptomatic.

## Figures and Tables

**Table 1:** Attack rates of listeriosis among pregnant women exposed to contaminated cantaloupe, estimated using three methods, Colorado, 2011.

Method (equation)^a^	Pregnancy-associated listeriosis	Estimated exposed women^b^	Estimated attack rate (per 10,000 exposed pregnant women)
(1) Colorado cantaloupe distributed nationally (3)	1	6,500	1.5
(2) Grocery store chains (4)	1	12,000	0.9
(3) Pregnant residents and cantaloupe consumption (5)	1	7,200	1.4

^a^See Section 2.4 for explanations and equations.

^b^Calculated using unrounded estimates; results rounded to two significant figures.
